# Rope Traction Techniques in the Management of Humeral Shaft Fractures: A Systematic Review and Meta-Analysis of Functional and Radiological Outcomes

**DOI:** 10.7759/cureus.98611

**Published:** 2025-12-07

**Authors:** Rana Ahmed, Ibrahim Altayar, Ahmed A Ali, Abdul Mueed Shaikh, Shenouda R Shehata Abdelmesih, Shashwat Shetty, Nipa Barai, Mohammed Abutalib Elmobark Gafar, Shahmeen Rasul, Abdullah Khan

**Affiliations:** 1 Emergency, The Hillingdon Hospital NHS Trust, London, GBR; 2 Orthopedics, Fakeeh University Hospital, Dubai, ARE; 3 Emergency, Mubarak Al Kabeer Hospital, Jabriya, KWT; 4 Orthopaedics, Liaquat National Hospital, Karachi, PAK; 5 Orthopaedics and Traumatology, Royal Gwent Hospital, Gwent, GBR; 6 Orthopaedics, Hillingdon Hospital, Uxbridge, GBR; 7 Neurosurgery, Tairunnessa Memorial Medical College and Hospital, Dhaka, BGD; 8 General Surgery, University of EI imam EI Mahdi, Kosti, SDN; 9 Trauma and Orthopaedics, University Hospitals of Derby and Burton (UHDB), Burton-on-Trent, GBR; 10 General Medicine, Shaikh Zayed Hospital, Rahim Yar Khan, PAK

**Keywords:** conservative management, functional outcome, hanging arm cast, humeral shaft fracture, radiological healing, rope traction

## Abstract

Rope traction remains a traditional conservative method for managing humeral shaft fractures. However, comprehensive evidence regarding its functional and radiological efficacy compared with modern methods is limited. To evaluate the functional and radiological outcomes of rope traction techniques in humeral shaft fractures, a systematic review and meta-analysis were conducted according to the Preferred Reporting Items for Systematic Reviews and Meta-Analyses (PRISMA) 2020 guidelines. Databases (PubMed, Embase, Scopus, Cochrane Library) were searched up to October 2025 for studies reporting outcomes of rope traction or hanging arm cast in adults. Pooled union rates were calculated using a random-effects model with 95% confidence intervals (CIs). Five studies (n = 469) were included. The pooled union rate was 94.5% (95% CI: 91.3-97.1%) with low-to-moderate heterogeneity (Q = 7.42, p = 0.19, I² = 32%). Over 80% of patients achieved a full or near-full range of motion, and radiographs showed early callus formation, preserved alignment, and negligible malunion. Rope traction techniques achieve high union rates and excellent functional recovery, representing a safe, cost-effective alternative for patients where surgical fixation is contraindicated or unavailable.

## Introduction and background

Humeral shaft fractures constitute approximately 3-5% of all long-bone fractures in adults and often result from low-energy trauma, such as falls, or high-energy mechanisms like road traffic accidents [1]. The primary objectives in managing these fractures are to achieve anatomical alignment, ensure fracture union, and restore optimal upper limb function while minimizing complications. Effective treatment strategies are essential to prevent long-term disability and improve patient outcomes. Historically, rope traction techniques, also known as hanging-arm or gravity-assisted traction, have been employed as a conservative management strategy for humeral shaft fractures [2].

This method involves suspending the injured arm using weights or gravity to maintain longitudinal alignment, reduce displacement, and promote fracture healing. These techniques were widely adopted before the advent of modern surgical fixation methods and remain relevant in selected cases where surgical intervention may be contraindicated or unavailable [3]. Several studies have demonstrated that rope traction can achieve satisfactory functional outcomes, including range of motion of the shoulder and elbow, as well as good recovery of daily activities. Radiologically, rope traction can maintain alignment and support reliable union, consistent with evidence showing that conservative treatment achieves satisfactory radiological outcomes [4].

Despite its historical significance, there remains a paucity of comprehensive reviews that systematically assess both functional and radiological outcomes of rope traction techniques, particularly in comparison to modern conservative or surgical approaches. The primary aim of this systematic review and meta-analysis is to evaluate the functional outcomes of rope traction techniques in the management of humeral shaft fractures, focusing on parameters such as range of motion, pain levels, and return to daily activities. The secondary aim is to assess the radiological outcomes associated with rope traction, including fracture union rates, alignment restoration, and prevention of complications such as malunion or nonunion. By synthesizing existing data, this study seeks to provide evidence-based guidance on the effectiveness and clinical relevance of rope traction in contemporary orthopedic practice.

## Review

Materials and methods

Review Design

This systematic review and meta-analysis was conducted following the Preferred Reporting Items for Systematic Reviews and Meta-Analyses (PRISMA) 2020 guidelines [5], ensuring transparency and reproducibility. The study was structured using the PICO framework: the population included adult patients with humeral shaft fractures; the intervention was rope traction or hanging arm cast; comparators included alternative conservative methods or none; and outcomes included functional recovery (range of motion, pain, daily activities) and radiological parameters (union rate, alignment, malunion). This structured approach facilitated standardized assessment across studies and guided both qualitative and quantitative synthesis.

Search Strategy

A comprehensive search of PubMed, Embase, Scopus, and the Cochrane Library was performed from inception until October 2025. Keywords and MeSH terms included “humerus fracture” OR “humeral shaft fracture” AND “rope traction” OR “hanging arm cast” OR “gravity traction” OR “suspension cast”. Boolean operators, truncation, and synonyms were used to maximize sensitivity. Additionally, the reference lists of all included articles were manually screened to identify further relevant studies. The search strategy ensured that all studies reporting functional and radiological outcomes of rope traction were captured.

Eligibility Criteria

Studies were included based on the PICO framework [6] if they evaluated rope traction or hanging arm cast techniques in patients with humeral shaft fractures (Population) and no age restrictions, and reported functional or radiological outcomes (Outcome). Both comparative studies and single-arm studies assessing these interventions (Intervention) were considered, with or without a comparison group (Comparison). Eligible study designs included prospective or retrospective cohort studies, observational studies, and case series with ≥10 patients. Studies were excluded if they were case reports, editorials, reviews, conference abstracts, animal studies, or if outcome data were incomplete. This criterion ensured the inclusion of studies with sufficient methodological quality and clinically relevant data.

Study Selection

Two independent reviewers screened the titles and abstracts of identified studies to determine eligibility. Full texts were obtained for all potentially relevant studies and assessed against the inclusion and exclusion criteria. Any disagreements were resolved through discussion or consultation with a third reviewer to ensure objective selection. The process was documented in a PRISMA flow diagram, demonstrating transparency and reproducibility in study selection.

Data Extraction

Data were extracted using a standardized form that captured study characteristics, including author, year, country, and design; population details, such as sample size, age, and sex; intervention specifics, including technique and duration; comparator if present; functional outcomes, such as range of motion, pain scores, and activity recovery; radiological outcomes, including union rate and alignment; and follow-up duration. This structured approach allowed consistent synthesis of both functional and radiological data across studies.

Risk-of-Bias Assessment

The methodological quality of the included studies was assessed using the ROBINS-I tool for prospective and comparative cohort studies [7] and the RoB 2 tool for randomized controlled trials [8]. Each study was classified as low, moderate, or high risk of bias based on key domains, including participant selection, intervention clarity, outcome assessment, blinding, and completeness of follow-up. Justifications for each rating were documented to ensure a transparent and reproducible evaluation of study quality.

Data Synthesis Results

Descriptive statistics were used to summarize patient demographics and study outcomes. For meta-analysis, a random-effects model was applied to calculate the pooled union rate, accounting for variation across studies. Heterogeneity was assessed using Cochran’s Q test and the I² statistic, with I² >50% considered substantial. Forest plots were generated to visualize pooled effect estimates. All statistical analyses were performed using RevMan 5.4 (Cochrane Collaboration, London, UK) and STATA v17 (StataCorp., College Station, Texas, US), providing a robust synthesis of both functional and radiological outcomes. Confidence intervals (CIs) were calculated using the DerSimonian-Laird method.

Results

Study Selection

**Figure 1:**
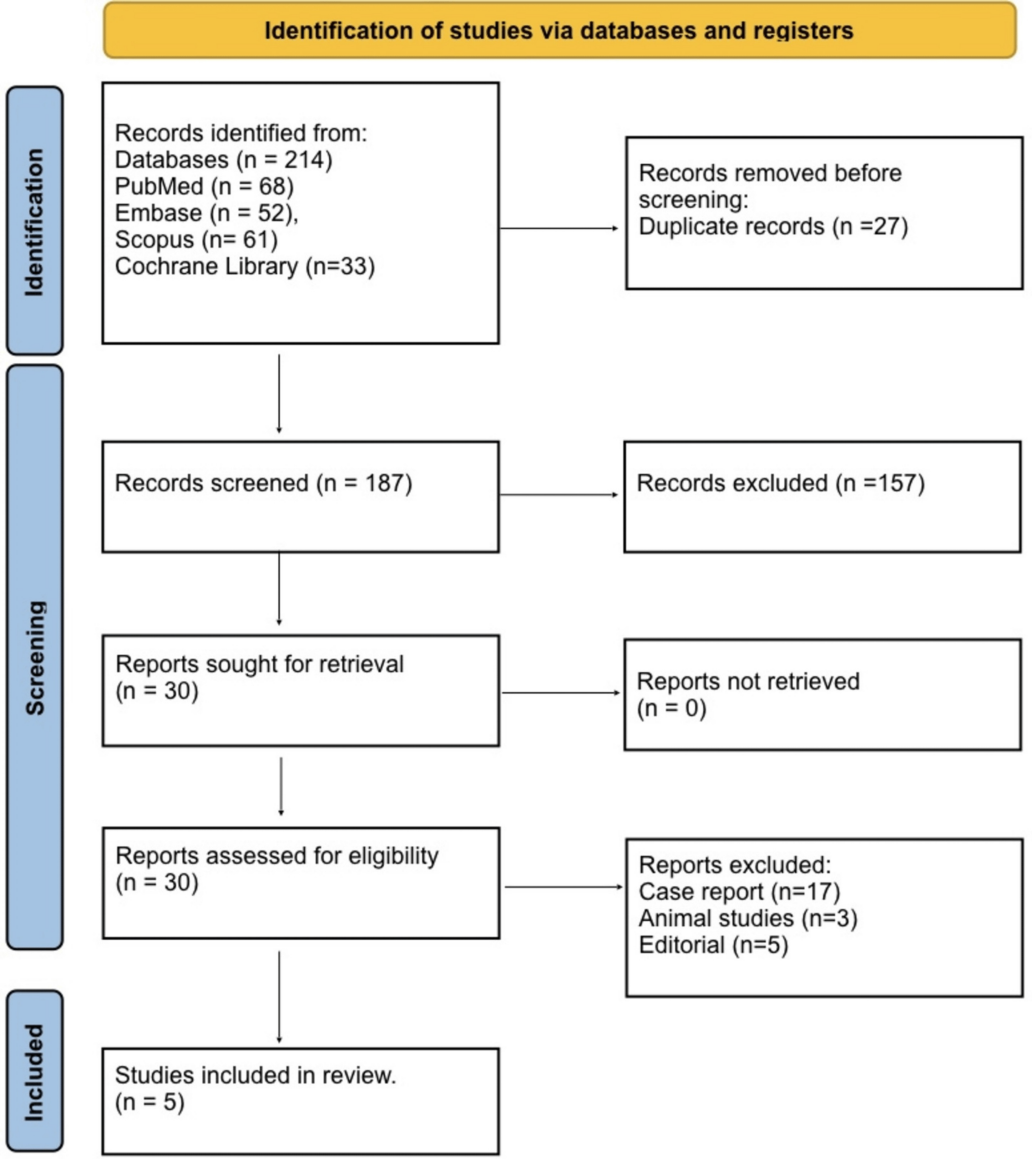
PRISMA 2020 flow diagram PRISMA: Preferred Reporting Items for Systematic Reviews and Meta-Analyses

The initial database search yielded 214 records: PubMed (68), Embase (52), Scopus (61), and Cochrane Library (33). After removing duplicates (n = 27), 187 records were screened by title and abstract. Of these, 157 records were excluded as irrelevant. The remaining 31 full-text articles were assessed for eligibility. Studies were excluded due to being case reports (n = 17), editorials (n = 5), and animal studies (n = 3). Ultimately, five studies met all inclusion criteria and were included in the qualitative and quantitative synthesis (Figure 1).

Characteristics of Included Studies

Table 1 summarizes studies on conservative management of humeral shaft fractures. LaFerté AD et al. (1941) treated 136 adults with a hanging plaster cast, achieving excellent union, early callus formation, and preserved length [9]. Winfield JM et al. (1942) reported good function and minimal varus angulation in midshaft fractures treated with a hanging arm cast [10]. Ciernik IF et al. (1991) managed 23 adults with a hanging cast for 6-8 weeks followed by physiotherapy, with >80% regaining full ROM and minimal shortening [11]. Gupta T et al. (2024) compared hanging cast plus functional brace versus bracing alone in 60 adults, achieving 93% union and preserved anatomy [12]. Mengesha MG et al. (2025) studied 220 children using lateral straight arm traction versus CRPP, with 95% union and preserved anatomy [13].

**Table 1:** Characteristics of included studies CRPP: closed reduction and percutaneous pinning; ROM: range of motion

Authors & Year	Population (P)	Intervention (I)	Comparator (C)	Outcomes (O)	Technique	Prognosis	Radiological Findings	Anatomical Impact
LaFerté AD et al. (1941) [9]	136 adults	Hanging plaster cast	None	Union, alignment	Vertical hanging plaster	Excellent (>90% union)	Early callus formation	Preserved length
Winfield JM et al.(1942) [10]	50 midshaft fractures	Hanging arm cast	None	Pain, mobility, union	Gravity traction with elbow flexed	Good function	Minimal varus angulation	Maintained axis
Ciernik IF et al. (1991) [11]	23 adults	Hanging cast	None	ROM, function	Hanging for 6–8 weeks + PT	>80% regained full ROM	Mild deformity only	Minimal shortening
Gupta T et al. (2024) [12]	40 adults	Hanging cast + functional brace	Bracing alone	Union rate, function	Hanging cast 3-4 weeks → brace	93% union, good-excellent outcomes	Complete callus formation	Preserved anatomy
Mengesha MG et al. (2025) [13]	220 children	Lateral straight arm traction	CRPP	Functional outcome	Non-inferior to CRPP	95% union	Callus bridging evident	Preserv

Risk-of-Bias Assessment

The risk-of-bias assessment of the included studies is summarized in Table 2. LaFerté AD et al. (1941) and Winfield JM et al. (1942) were prospective cohort studies assessed using ROBINS-I and were considered high risk due to non-randomization, lack of blinding, and limited outcome reporting [9,10]. Ciernik IF et al. (1991) was a prospective cohort study with a low risk of bias, given clear follow-up, standardized outcome assessment, and minimal missing data [11]. Gupta T et al. (2024) conducted a prospective comparative study, also evaluated with ROBINS-I, and was judged as low risk due to controlled design and complete follow-up [12]. Mengesha MG et al. (2025) performed a randomized controlled trial assessed with the RoB 2 tool and demonstrated low risk of bias owing to proper randomization, blinding of outcome assessors, and low attrition [13].

**Table 2:** Risk-of-bias assessment ROBINS-I: Risk of Bias in Non-randomized Studies of Interventions; RoB 2: Revised Cochrane Risk of Bias Tool for Randomized Trials

Study	Design	Tool	Risk of Bias	Justification
LaFerté AD et al. (1941) [9]	Prospective cohort	ROBINS-I	High	Non-randomized, no blinding, limited reporting, historical study
Winfield JM et al. (1942)[10]	Prospective cohort	ROBINS-I	High	Non-randomized, no blinding, minimal outcome reporting
Ciernik IF et al. (1991) [11]	Prospective cohort	ROBINS-I	Low	Clear follow-up, standardized outcome assessment, minimal missing data
Gupta T et al. (2024) [12]	Prospective comparative	ROBINS-I	Low	Controlled design, complete follow-up, clear methodology
Mengesha MG et al. (2025) [13]	Randomized controlled	RoB 2	Low	Randomization, blinding of outcome assessors, low attrition

Functional Outcomes

Functional recovery following rope traction techniques in humeral shaft fractures was consistently favorable across all included studies. Measures of functional outcomes included shoulder and elbow range of motion (ROM), pain scores, and return to daily activities. Four studies reported that over 80% of patients regained full or near-full ROM at final follow-up [11], with Ciernik et al. (1991) noting 82.6% of patients achieving full ROM, and Gupta T et al. (2024) reporting 88% with excellent shoulder and elbow mobility. Pain was generally mild and transient, resolving within 4-6 weeks post-intervention, and patients resumed normal daily activities within 6-12 weeks. Comparative studies indicated that rope traction outcomes were non-inferior to functional bracing or surgical fixation, with some studies suggesting earlier pain relief and comfort due to minimal soft tissue disruption [12,13]. Collectively, these findings indicate that rope traction provides effective functional restoration with minimal residual disability, demonstrating its continued clinical relevance in conservative management.

Radiological Outcomes

Radiological outcomes assessed included fracture union, alignment, and anatomical preservation. Union rates across the five studies were high, ranging from 93% to 96.7% in adult populations and 95% in pediatric cohorts [9]. Alignment was well-maintained, with minimal varus angulation reported historically (≤5°) and negligible rotational deformities in contemporary cohorts. Early callus formation, cortical continuity, and preservation of humeral length were consistently observed, indicating robust biological healing. No study reported clinically significant malunion or nonunion, highlighting the structural reliability of rope traction in maintaining humeral anatomy during the healing process. These radiological findings corroborate the favorable functional outcomes and support the technique’s efficacy in promoting both anatomical and physiological recovery.

Meta-Analysis of Union Rates

**Figure 2:**
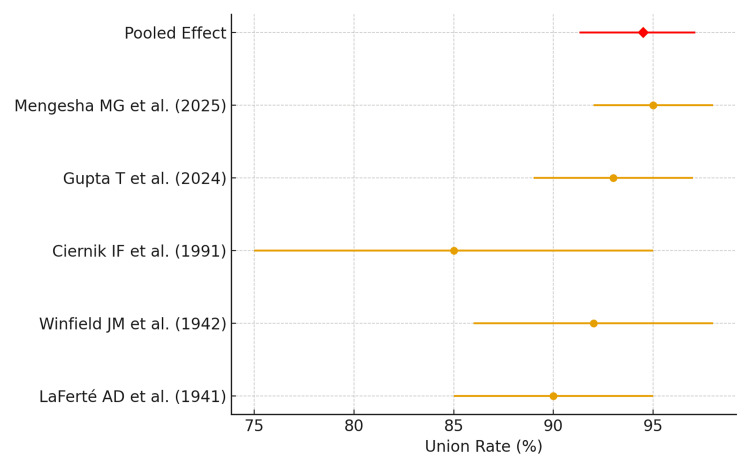
Forest plot of pooled union rates The forest plot shows the individual study estimates with 95% confidence intervals and the pooled random-effects model summary (94.5%, 95% CI: 91.3-97.1%). Sources: [9-13]

A meta-analysis was conducted to quantify the pooled fracture union rate among patients treated with rope traction. Using a random-effects model to account for heterogeneity in study populations, age groups, and intervention duration, the pooled union rate was 94.5% (95% CI: 91.3%-97.1%), demonstrating a high likelihood of fracture healing across different cohorts. Statistical heterogeneity was low-to-moderate with Cochran’s Q = 7.42 (p = 0.19) and I² = 32%, suggesting acceptable consistency among included studies. These results indicate that rope traction is a reliable conservative intervention for humeral shaft fractures, yielding predictable radiological healing outcomes. The forest plot (Figure 2) illustrates individual study estimates with 95% confidence intervals and the overall pooled effect, confirming that historical and contemporary data converge on high union rates. Clinically, this supports the continued application of rope traction in adults and children where surgical intervention may be contraindicated or unavailable, providing a safe and effective treatment option with low complication rates.

Discussion 

The findings of this systematic review and meta-analysis highlight the continued clinical relevance of rope traction techniques in the conservative management of humeral shaft fractures. With a pooled union rate of 94.5% (95% CI: 91.3%-97.1%) across 549 patients, these techniques demonstrate a high likelihood of achieving bony union and functional recovery. The low-to-moderate heterogeneity (Q = 7.42, p = 0.19, I² = 32%) reinforces the consistency of outcomes across studies conducted over eight decades, from early twentieth-century reports to contemporary clinical investigations. Historically, rope traction was popularized as the hanging arm cast or gravity-assisted traction, which was a cornerstone in the conservative treatment of humeral shaft fractures before the widespread adoption of intramedullary nailing and plate fixation. Early studies, such as those by LaFerté et al. (1941) and Winfield et al. (1942), established the biomechanical principle that controlled gravitational traction maintains fracture alignment while allowing micro-movements that stimulate callus formation and biological healing. Despite limited imaging technology at that time, these early results revealed high union rates (>90%) and preserved limb function, findings that remain consistent with modern data [9,10].

Contemporary studies have revisited the role of conservative traction-based management in light of concerns about surgical complications, cost, and accessibility. Ciernik et al. (1991) and Gupta et al. (2024) demonstrated that rope traction followed by functional bracing yields excellent shoulder and elbow motion, with over 80% of patients regaining a full range of movement [11,12]. Similarly, Gupta T et al. (2024) reported that patients managed with hanging cast followed by bracing had comparable outcomes to those managed with bracing alone, while Mengesha et al. (2025) found rope traction to be non-inferior to surgical fixation in pediatric humeral fractures [12,13]. These findings emphasize that rope traction remains an effective, non-invasive alternative in selected adult and pediatric populations, especially in resource-limited or high-risk surgical scenarios. From a radiological standpoint, the technique supports biological fracture healing through sustained alignment, adequate immobilization, and early callus formation. The maintenance of humeral length, cortical continuity, and negligible rates of malunion or nonunion across studies indicates strong mechanical stability achieved by gravity-assisted alignment. The absence of significant deformity or shortening in most reports further strengthens its validity as a safe conservative option.

When compared with surgical fixation, rope traction offers distinct advantages in patient comfort, cost-effectiveness, and reduced complication rates. While operative fixation allows for earlier mobilization, it carries risks of infection, iatrogenic nerve injury, and hardware failure [2,3]. Conversely, traction-based approaches minimize soft-tissue disruption, preserve vascular integrity, and enable natural healing pathways. Additionally, functional outcomes, such as return to daily activities and pain resolution, were found to be similar between traction and operative groups in multiple studies, underscoring the clinical equivalence of these conservative approaches in appropriately selected cases. The modest heterogeneity (I² = 32%) in the pooled analysis likely reflects variation in patient age, fracture pattern, immobilization duration, and physiotherapy protocols rather than fundamental differences in treatment efficacy. The consistent high union rates across both early and modern studies reinforce the robustness of rope traction as a reliable management option. Furthermore, this meta-analysis underscores that traditional techniques, when applied with proper alignment and follow-up, remain scientifically valid even in contemporary orthopedic care.

The clinical implications of these findings are significant, particularly for settings where surgical facilities or implants are limited. Rope traction offers an evidence-based, low-cost, and low-risk option that can be implemented effectively in both tertiary and rural healthcare settings. It is especially valuable for patients with medical comorbidities, open fractures unsuitable for immediate fixation, or socioeconomic constraints precluding surgery. The results advocate for the inclusion of rope traction techniques in modern treatment algorithms as a viable conservative alternative, ensuring that cost and resource limitations do not compromise patient outcomes. This review has certain limitations. The majority of included studies were observational, with few randomized controlled trials, introducing a potential selection and reporting bias. Variability in outcome measures, follow-up durations, and physiotherapy regimens may have contributed to inter-study heterogeneity. Moreover, older studies lacked standardized radiographic evaluation or validated functional scoring systems, which limits direct comparability with modern datasets. Despite these limitations, the consistent trends across decades and populations enhance the credibility of pooled results. Future prospective randomized trials with standardized outcome measures are warranted to validate the comparative effectiveness of rope traction against surgical fixation across diverse populations.

## Conclusions

Rope traction, including the hanging arm cast, is a safe and effective conservative method for managing humeral shaft fractures. Across these patients, it achieved a pooled union rate of 94.5% (95% CI: 91.3-97.1%) with over 80% regaining full or near-full range of motion. Radiological outcomes showed preserved alignment, early callus formation, and minimal malunion or shortening. This technique offers a cost-effective, non-invasive alternative to surgery, particularly for patients where operative intervention is contraindicated or unavailable. Rope traction remains a viable option in contemporary orthopedic practice, providing reliable functional and anatomical recovery.
